# The Contribution of Learning and Mental Health Variables in First-Year Students' Profiles

**DOI:** 10.3389/fpsyg.2021.627118

**Published:** 2021-04-21

**Authors:** Fotios S. Milienos, Christos Rentzios, Leen Catrysse, David Gijbels, Sofia Mastrokoukou, Claudio Longobardi, Evangelia Karagiannopoulou

**Affiliations:** ^1^Panteion University, Athens, Greece; ^2^University of Ioannina, Ioannina, Greece; ^3^University of Antwerp, Antwerp, Belgium; ^4^University of Milano Bicocca, Milan, Italy; ^5^University of Turin, Turin, Italy

**Keywords:** self-regulation, higher education, achievement, GPA, student mental health

## Abstract

International studies focus on the successful transition into higher education, which is considered crucial for both the students and the educational institution in the context of students' learning and adjustment in higher education. The aim of the current study was to identify student profiles that include cognitive, metacognitive, and motivational aspects of learning, but also aspects of resilience, emotion dysregulation, and anxiety. The sample consists of 316 Greek undergraduate students (18.7% males and 81.3% females). The results showed four different (meta)-cognitive-emotional learner profiles: the emotionally stable and highly adaptive learner; the emotionally dysregulated and at risk learner; the emotionally dysregulated and highly adaptive learner; the emotionally stable and at risk learner. Emotionally dysregulated and at risk learner has a lower GPA than the emotional stable and highly adaptive learner, the emotionally dysregulated and highly adaptive learner and the emotionally stable and at risk learner.

## Introduction

International studies focus on the successful transition into higher education, which is considered crucial for both the students and the educational institution (Tinto, 2015). Over the last decade, the Organization for Economic Co-operation and Development has reported that approximately one-third of students entering higher education will not graduate (OECD, 2013). The majority of students' withdrawals occur during the first year of studies (Hultberg et al., 2008; Webb and Cotton, 2018; Gilar-Corbi et al., 2020), a year that is considered extremely critical for the overall success in undergraduates' studies (Perry et al., 2001). Thus, this initial phase of higher education sets the stage for either to earn a degree or dropout from university (Tinto, 1993; Díaz Mujica et al., 2019; Nicoletti, 2019) and still remains a major political concern in Europe (Vossensteyn et al., 2015).

Transition is not thought of as a single event but rather regarded as an on-going process that is repeated over time (Tett et al., 2017). Students seem to have a difficulty to understand the differences between studying at a university and studying at an upper secondary school or the demands of the university level teaching-learning environment (Haarala-Muhonen et al., 2017). This transition may become an especially stressful period for many freshman students (Longobardi et al., 2016, 2019; Coertjens et al., 2017), as they have to deal with a number of serious challenges, such as the need for developing novel learning patterns, and also the adaptation of the already existing learning strategies in the new academic environment (Vermunt, 2005; Gasevic et al., 2017). In addition, recent studies report students' difficulties in academic adjustment that are mainly due to ineffective learning strategies and unsatisfactory self-regulation (lack of ability in monitoring learning progress, difficulty adapting their behavior in the demands of the new learning situations and the new learning context) (Hoffait and Schyns, 2017).

In this line of thinking, the first year of studies in university appears to play an important role in students' future academic achievement and well-being, and consequently in their future professional success and their personal development (Leese, 2010; Trautwein and Bosse, 2017). Even though the predictive power of cognitive factors has been extensively studied, the investigation of a combination of non-cognitive factors such as self-regulation, motivation, and anxiety (Fonteyne et al., 2017; Willems et al., 2018) along with mental health and personality variables (Schneider and Preckel, 2017; Schaeper, 2019) should also be included as they seem to influence this crucial period in students' life.

### Self-Regulation and Motivation

The student learning strategies and motivation have been found to be related to learning outcomes such as academic performance and students' dropout (Robbins et al., 2006; Vanthournout et al., 2012; Casanova et al., 2018). Learning strategies are viewed as cognitive-processing learning and regulation strategies adopted by students during their learning activities (Vermunt and Donche, 2017). Processing strategies include deep, stepwise, and concrete processing activities; relating, structuring, and critical processing are considered as deep processing, whereas memorizing and analyzing are thought of as a stepwise approach in processing. Concrete processing is linked to a vocation orientation (Vanthournout et al., 2012). Regulation strategies refer to activities that students adopt to harness their cognitive processing strategies (Schunk and Zimmerman, 2012). Self-regulation is associated with higher achievement, whereas lack of regulation is related to lower academic achievement (Vermunt, 2005; Ramli et al., 2018).

Study motivation is also considered another important predictor of academic success and dropout (Bailey and Phillips, 2016; Rump et al., 2017). Motivation is viewed in terms of self-efficacy, which can be defined as students' judgments and beliefs of their capabilities to perform a task in the course (Zusho et al., 2003). For example, self-efficacious students are able to achieve better in academic tertiary because they give more importance to performance and mastery goals (Komarraju and Nadler, 2013). Moreover, the positive interaction between self-regulated learning and motivation is well-established in relative research. Self-regulated learners who show strong self-efficacy are less likely to procrastinate as they seem to control their motivation (Katz et al., 2014). Additionally, Klassen et al. (2008) found that students who procrastinate lack the confidence needed to apply useful strategies in completing tasks. It is clear that when students combine self-regulation skills and a strong sense of motivation (self-efficacy), may potentially reduce procrastination and facilitate higher academic performance (Burnam et al., 2014), a fact that emphasizes the role of self-efficacy as a factor underpinning procrastination (Steel, 2007; Arias-Chávez et al., 2020).

### Anxiety and Emotion Dysregulation

Emotions and the way students regulate their emotions during transition play an important role in students' academic life (Srivastava et al., 2009). On the other hand, emotion dysregulation is considered as a difficulty in regulating emotions during stressful situations (Semplonius and Willoughby, 2018) interfering with individuals' targeted goals (Thompson, 2019) and playing a major role in college life (Fischer et al., 2007). In a recent quantitative study, Wagner and Brahm (2017) recognize that students who are afraid of failing their courses have a lower possibility of advancing toward their first year. Moreover, the difficulties in emotion regulation were negatively correlated with GPA (Hartman et al., 2017), and may lead freshmen to severe mental health issues (e.g., depression) along with problems with social satisfaction and well-being (Tamir et al., 2007; Kneeland and Dovidio, 2019).

During the first year of studies students are confronted with new tasks, demands, and competitive environments that cause high levels of stress and anxiety (DeBerard et al., 2004; Leese, 2010; Respondek et al., 2017). One-third of the university student population experiences symptoms of anxiety and depression (American Psychiatric Association, 2013). Students that experience high levels of anxiety are less efficient using less self-regulated learning strategies (Pintrich, 2004) and characterized by low levels of well-being, self-acceptance, and self-control (Hembree, 1988). Furthermore, anxiety is positively correlated with delays in starting or completing tasks on time and meeting deadlines, thus possibly leading students to procrastinate more often (Chang, 2014).

### Procrastination and Resilience

Procrastination can be defined as “the voluntary delay of an intended and necessary and/or [personally] important activity, despite expecting potential negative consequences that outweigh the positive consequences of the delay” (Klingsieck, 2013 p. 26). Almost, all students occasionally procrastinate and approximately every second student regularly procrastinates in one or another domain of their studies (Steel, 2007). Contemporary findings indicate that 30–60% of students regularly postpone completing their educational tasks. Moreover, it is closely associated with students' retention, academic achievement, and dropout intentions (Kim and Seo, 2015; Bäulke et al., 2018) with some factors like emotion regulation, motivation, and self-regulation appear to buffer its negative effect (Dunn, 2014; Eckert et al., 2016). In addition, procrastination seems to result from a complex array of factors that “work” against it, namely, emotional, motivational, and cognitive factors. A recent study has revealed an integrated picture of these variables in relation to procrastination, “showing the way” for future research. Nevertheless, procrastination is thought of as a pivotal factor that may be detrimental to student's academic achievement and pace of study.

Resilience has been acknowledged as a capability to bounce back and recover from stressful circumstances in order to adjust to the environment (Smith et al., 2008; Turner et al., 2017). It is usually studied as a dispositional trait linked to personality (Sagone and De Caroli, 2014) that acts as a protective factor against extreme stress and adversity, while the individual maintains normal physiological and physical functioning (Russo et al., 2012). Previous researches in a university sample have noted that resilience is negatively correlated to stress (Ahern and Norris, 2011; Shi et al., 2015) in addition to promoting students' well-being (Turner et al., 2017). Moreover, it has been considered as a skill that assists university students in their transition to higher education (DeRosier et al., 2013), and is usually involved in helping to understand students' retention and success (Cotton et al., 2017). In relation to procrastination, high resilient individuals have been found to show fewer procrastinative behaviors at all stages of the career decision-making process. It is without a doubt that in today's competitive and demanding university context, resilience is critical and should be taken into account, mainly because it works as a buffer against procrastination.

### Aim of the Study

In the context of students' learning and adjustment in higher education, the aforementioned studies have examined and analyzed the contribution of each factor separately. It remains of high importance to understand that the adoption of particular learning patterns, students' motivation, and non-cognitive characteristics are based on a dynamic interaction between different factors and variables, focusing on the student him/herself. Moreover, the present study takes into account variables from fields that have not been studied in conjunction: resilience, emotion dysregulation, and anxiety (mental health field).

The current study answers the question regarding the way in which these specific factors could possibly interact cumulatively, influencing students' pace of study, procrastination and GPA.

Previous studies exploring first-year students' academic success have come up with two to four profiles (Haarala-Muhonen et al., 2017; Lindblom-Ylänne et al., 2017). However, as these studies do not include variables from different theoretical traditions, it would be interesting to explore whether mental health variables could also contribute to the formulation of clusters (Karagiannopoulou et al., 2019). Based on the above we have postulated the following research questions:

RQ1: Which different (meta) cognitive-emotional learner profiles can be identified for first-year students in higher education?

RQ2: How do these different (meta) cognitive-emotional learner profiles differ regarding GPA and success rate?

## Method

### Participants

The sample consists of 316 undergraduates (18.7% males and 81.3% females) studying in the School of Social Sciences in the University of Ioannina, Greece. In Greece, the gender ratio in Schools of Social Sciences is overwhelmingly in favor of women (Eurostat, 2018; Karagiannopoulou et al., 2018, 2020). The students anonymously completed the questionnaires in their classes before or during the break of the lecture. The teacher and the students had agreed on their contribution to the study in a previous class meeting with the research team. A written informed consent in compliance with the ethical regulations and guidelines established by the Ethics Committee of the University of Ioannina was obtained from all participants prior to the administration of the questionnaires, the completion of which lasted ~35 min on average.

### Instruments

The psychometric properties of ILS (Vermunt, 1994, 1998), MSLQ (Pintrich et al., 1991), RS, DERS-18 (Gratz and Roemer, 2004), DASS-21 (Lovibond and Lovibond, 1995), and PASS (Solomon and Rothblum, 1984) have been explored in previous studies and proved to be robust research instruments (Wagnild and Young, 1993; Vermunt and Vermetten, 2004; Komarraju and Nadler, 2013; Beiter et al., 2015; Kim and Seo, 2015; Skutch et al., 2019).

### Inventory of Learning Patterns for Students (ILS)

To measure students' learning strategies the Inventory of Learning patterns of Students (ILS) was administered (Vermunt, 1994, 1998; Vermunt and Donche, 2017). The version of ILS that we use consists of 47 items divided in two parts. The first part includes questions about processing strategies: deep (4 items, e.g., “I compare the conclusions drawn in different chapters”), stepwise (6 items, e.g., “I memorize definitions as literally as possible”), and concrete processing (3 items, e.g., “When I am studying a topic, I think of cases I know from my own experience that are connected to that topic”), and (ii) regulation strategies: self-regulation (5 items, e.g., “I add something to the subject matter from other sources”), external (5 items, e.g., “I study according to the instructions given in the study materials or provided by the teacher”), and lack of regulation strategies (4 items, e.g., “I notice that I have trouble processing a large amount of subject matter”), respectively.

The second part addresses questions about learning orientations: personal interest (5 items, e.g., “I do these studies because I like to learn and to study”), test oriented (5 items, e.g., “I view the choice I have made to enroll in higher education as a challenge”), vocation oriented (5 items, e.g., “The main goal I pursue in my studies is to prepare myself for a profession”), and ambivalent (5 items, e.g., “I doubt whether this is the right subject area for me”). Participants in the first part answer each item on a 5-point Likert scale ranging from 1 (almost never) to 5 (almost always). In the second part, each item is scored also on a 5-point Likert scale, ranging from 1 (disagree entirely) to 5 (agree entirely).

#### Strategies for Learning Questionnaire (MSLQ)

Motivated strategies for learning were measured with the Motivated Strategies for Learning Questionnaire MSLQ (Pintrich et al., 1991), one of the most widely used instruments for measuring students' self-regulated learning. For the purposes of our study, we use only the self-efficacy of learning and performance subscale (8 items, e.g., “I expect to do well in this class”). Each item is scored on a 5-point Likert scale, ranging from 1 (disagree entirely) to 5 (agree entirely).

#### The Resilience Scale (RS)

In order to measure resilience, the Resilience Scale was selected. RS is a self-reported measure of 25 items. Responses are summed to produce a total score. The participants are asked to state the degree to which they agree or disagree with each item on a 5-point Likert-type scale from 1 (disagree entirely) to 5 (agree entirely). All items are positively scored. The total scores thus range from 25 to 125 with higher scores reflecting higher resilience. Items examples are: “I have self-discipline” or “I can usually look at a situation in a number of ways.”

#### Difficulties in Emotion Regulation Scale (DERS-18)

DERS-18 is a short version of the original DERS (Gratz and Roemer, 2004) that has been recently developed by Victor and Klonsky (2016). It is used to evaluate various aspects of emotion regulation difficulties. It comprises 6 subscales, namely awareness (e.g., “I pay attention to how I feel”), clarity (e.g., “I have no idea how I am feeling”), goals (e.g., “When I'm upset, I have difficulty getting work done”), impulse (e.g., “When I'm upset, I become out of control”), strategies (e.g.,” When I'm upset, I believe that I'll end up feeling very depressed”), and non-acceptance (e.g., “When I'm upset, I feel guilty for feeling that way”). The items in DERS-18 are rated on a 5-point Likert scale ranging from 1 (I do this almost never) to 5 (I do this almost always). A higher score indicates greater emotion dysregulation.

#### Depression—Anxiety—Stress Scale (DASS-21)

The DASS-21 is a self-reported instrument that independently assesses three factors: depression, anxiety, and stress (Lovibond and Lovibond, 1995). For our study we use only the anxiety scale (7 items, e.g., “I felt scared without any good reason”). Anxiety scale is scored on a 5-point Likert scale ranging from 1 (I do this almost never) to 5 (I do this almost always). A higher score is indicative of high level of anxiety.

#### Procrastination Assessment Scale Student (PASS)

Procrastination was assessed with the PASS (Solomon and Rothblum, 1984), a self-reported measure that evaluates the frequency of students' procrastination. Participants are asked to answer questions regarding procrastination in a 5-point Likert scale in six academic domains: writing a term paper, studying for an exam, keeping up weekly reading assignments, academic administrative tasks, attendance tasks, and school activities in general. Answers range from 1 (I do this almost never) to 5 (I do this almost always) with the highest score indicating higher procrastination.

#### Pace of Study

Students' pace of study was assessed by (self-reported) Grade Point Average (GPA) and courses success rate. Success rate is computed as the proportion of the number of courses they had passed until the time of data collection and then, to the total number of courses they have already attended.

### Data Analysis

Data analysis starts with some descriptive statistics and continues with assessing the latent structure and reliability of the instruments used in our study; confirmatory factor analysis, average variance extracted, and Cronbach's reliability coefficient were among the basic tools for this purpose. After, a cluster analysis is carried out (using the R-software) for classifying students into homogeneous groups according to their responses to (20 subscales): (i) processing strategies (three subscales; deep, stepwise and concrete processing), (ii) regulation strategies (three subscales; self-regulation, external, and lack of regulation strategies), (iii) learning orientations (four subscales; personal interest, test oriented, vocation oriented, and ambivalent, (iv) motivation and learning strategies (one subscale; self-efficacy), (v) resilience scale, (vi) emotion regulation difficulties (six subscales; awareness, clarity, goals, impulse, strategies, and non-acceptance, (vii) depression-anxiety-stress (one subscale; anxiety), and (viii) procrastination (one subscale; procrastination). The role of the derived clustering on GPA and success rate was subsequently examined.

The decision about the underlying number of clusters was taken by using the package “NbClust” (Charrad et al., 2014) provided by R-project (R Core Team, 2019); specifically, five distance measures (euclidean, maximum, manhattan, Canberra, and minkowski) and six clustering methods (kmeans, ward.D, ward.D2, single, complete, and average) were taken into account, evaluating any solution with number of clusters ranging from 2 to 10. Therefore, for each combination of distances and methods (30 in total), one optimal clustering solution was provided (based on 30 indices and the majority rule provided by this package); in order to determine the best clustering, we computed the proportion of statistically significant differences among cluster means (for each of the 30 solutions) on the 20 subscales (using Kruskal-Wallis test, i.e., functions “kruskal.test” and “kruskalmc”; see also Ch. 8 in Siegel and Castellan, 1988). It is necessary to mention that the above analysis was carried out on the standardized factor scores, computed by the confirmatory factor analysis and the empirical Bayes modal approach found in function “lavPredict” (see e.g., Rosseel, 2012).

Furthermore, the differences among clusters were also explored by the multivariate analysis of variance techniques (MANOVA), while discriminant analysis was used to assess the accuracy of classification.

## Results

Results from the Confirmatory Factor Analysis (CFA; the parameter estimation was based on the weighted least squares method) can be found in Table 1; note that the fit of the models on our data is assessed by the next indices (see, e.g., Raykov and Marcoulides, 2006; Kline, 2011): Comparative Fit Index (CFI), Normed Fit Index (NFI), Goodness-of-Fit Index (GFI), Adjusted Goodness-of-Fit Index (AGFI), Tucker-Lewis Index (TLI), Root Mean Square Error Approximation (RMSEA) (providing the *p*-value for testing the hypothesis: H_0_: RMSEA≤0.05 vs. H_1_: RMSEA>0.05) and Standardized Root Mean square Residual (SRMR). It is necessary to mention that some covariances between residual/error terms associated with indicators only from the same subscales, have been set not equal to zero. Therefore, it can be seen (Table 1) that most of the indices are found in acceptable range of values because most of AGFI, TLI, NFI, GFI, and CFI are larger than 0.90, while RMSEA and SRMR are quite small (for seven out of eight cases, H_0_: RMSEA≤0.05 is not rejected at 0.05 significance level). Extra caution must be paid to Motivation, Regulation Strategies and RS because of some unacceptable indices values. Note also that the last row of Table 1 includes the mean values of R-squares from all items included in the respective scales; RS and PASS (recall that only one factor is assumed for each of the two scales) have the smallest values, whereas DERS and DASS the largest ones.

**Table 1:** The CFA on the instruments used in our study.

	**Cognitive learning strategies (*ILS*)**	**Regulation strategies (*ILS*)**	**Motivation (*ILS*)**	**MSLQ**	**DERS**	**DASS**	**PASS**	**Resilience**
CFI	0.958	0.941	0.881	0.983	1.000	1.000	0.968	0.926
NFI	0.913	0.870	0.813	0.957	0.974	0.986	0.947	0.848
TLI	0.947	0.927	0.860	0.976	1.014	1.011	0.949	0.920
GFI	0.978	0.967	0.925	0.979	0.986	0.994	0.981	0.931
AGFI	0.967	0.953	0.903	0.963	0.981	0.987	0.964	0.918
RMSEA	0.051	0.047	0.066	0.045	0.000	0.000	0.065	0.050
*p*-value^*^	(0.442)	(0.632)	(0.001)	(0.590)	(1.000)	(0.990)	(0.068)	(0.486)
SRMR	0.065	0.061	0.083	0.065	0.047	0.044	0.067	0.077
R-square^**^	0.366	0.353	0.323	0.363	0.546	0.451	0.264	0.187

* *The p-value for testing the hypothesis: H_0_:RMSEA≤0.05 vs. H_1_:RMSEA>0.05*.

** *The mean value of R-square from all subscales included in the respective scale*.

Table 2 includes Cronbach's alpha, Average Extracted Variance (AVE) and mean values for the 20 subscales; Cronbach's alpha ranges from 0.547 (Awareness) to 0.849 (Goals) and two out of twenty subscales do not meet Fornell-Larcker criterion (i.e., square root of AVE for each of the latent factors is greater than the correlations with any other latent variable; (Fornell and Larcker, 1981).

**Table 2:** Cronbach's alpha, average extracted variance (AVE) and descriptive statistics, for the 20 subscales.

	**Subscales (number of items)**	**Alpha**	**AVE**	**Mean (std)**
Cognitive learning strategies (*ILS*)	Deep (4)	0.672	0.331^*^	10.67 (3.28)
	Stepwise (6)	0.749	0.340	19.84 (5.12)
	Concrete (3)	0.708	0.466	9.35 (2.63)
Regulation strategies (*ILS*)	Self (5)	0.705	0.324	13.09 (3.9)
	External (5)	0.635	0.342	17.71 (3.11)
	Lack (4)	0.733	0.364	8.79 (3.4)
Motivation (*ILS*)	Personal interest (5)	0.658	0.314^*^	19.32 (2.96)
	Test oriented (5)	0.689	0.310	18.70 (3.38)
	Vocation oriented (5)	0.676	0.420	19.68 (3.19)
	Ambivalent (5)	0.755	0.397	10.95 (3.82)
MSLQ	Self efficacy (8)	0.678	0.205	27.92 (4.9)
DERS	Awareness (3)	0.547	0.350	6.18 (2.16)
	Clarity (3)	0.841	0.645	6.33 (2.64)
	Goals (3)	0.849	0.655	8.97 (3.14)
	Impulse (3)	0.845	0.645	6.28 (2.98)
	Non-acceptance (3)	0.703	0.426	5.63 (2.43)
	Strategies (3)	0.770	0.530	5.75 (2.86)
DASS	Anxiety (7)	0.843	0.453	12.55 (5.63)
PASS	Procrastination (12)	0.779	0.331	29.35 (8.15)
RS	Resilience (25)	0.816	0.183	91.76 (9.69)

* *The Fornell-Larcker Criterion is not met*.

Pearson correlations can be found in Table 3; the largest positive correlations are between Strategies-Impulse (*r* = 0.79), Deep-Concrete (*r* = 0.782), Strategies- Goals (*r* = 0.773), Strategies-Non-Acceptance (*r* = 0.725), whereas the smallest negative correlations between Ambivalent-Personal Interest (*r* = −0.712), Ambivalent-Vocational Oriented (*r* = −0.533), and Resilience-Strategies (*r* = −0.49).

**Table 3:** Pearson correlation coefficient among subscales (factor scores).

	**Deep**	**Step-wise**	**Concrete**	**Self**	**External**	**Lack**	**Self efficacy**	**Awareness**	**Clarity**	**Goals**	**Impulse**	**Non-acceptance**	**Strategies**	**Anxiety**	**Personal interest**	**Test oriented**	**Vocation oriented**	**Ambivalent**	**Procrastination**
Deep	1	0.285^**^	0.782^**^	0.483^**^	−0.047	−0.088	0.369^**^	−0.179^**^	−0.210^**^	0.040	−0.006	−0.008	−0.042	0.003	0.355^**^	−0.021	0.112^*^	−0.216^**^	−0.069
Stepwise	0.285^**^	1	0.058	0.089	0.294^**^	−0.125^*^	0.149^**^	−0.138^*^	−0.087	−0.028	0.007	−0.011	−0.019	0.012	0.246^**^	0.203^**^	0.298^**^	−0.183^**^	0.088
Concrete	0.782^**^	0.058	1	0.480^**^	−0.171^**^	0.018	0.314^**^	−0.101	−0.119^*^	0.049	0.042	−0.026	−0.009	0.031	0.271^**^	−0.052	0.049	−0.164^**^	−0.042
Self	0.483^**^	0.089	0.480^**^	1	−0.125^*^	−0.203^**^	0.329^**^	−0.178^**^	−0.172^**^	−0.156^**^	−0.107	−0.053	−0.150^**^	0.070	0.351^**^	−0.035	0.050	−0.287^**^	−0.053
External	−0.047	0.294^**^	−0.171^**^	−0.125^*^	1	0.022	−0.049	−0.019	−0.076	0.041	0.005	−0.040	−0.024	0.012	0.043	0.158^**^	0.108	−0.040	0.147^**^
Lack	−0.088	−0.125^*^	0.018	−0.203^**^	0.022	1	−0.293^**^	0.129^*^	0.329^**^	0.339^**^	0.345^**^	0.217^**^	0.373^**^	0.116^*^	−0.214^**^	0.118^*^	−0.188^**^	0.316^**^	0.157^**^
Self efficacy	0.369^**^	0.149^**^	0.314^**^	0.329^**^	−0.049	−0.293^**^	1	−0.173^**^	−0.269^**^	−0.157^**^	−0.213^**^	−0.134^*^	−0.236^**^	−0.046	0.356^**^	0.054	0.190^**^	−0.339^**^	−0.146^**^
Awareness	−0.179^**^	−0.138^*^	−0.101	−0.178^**^	−0.019	0.129^*^	−0.173^**^	1	0.302^**^	0.028	0.033	0.144^*^	0.039	0.070	−0.116^*^	0.032	−0.036	0.153^**^	−0.021
Clarity	−0.210^**^	−0.087	−0.119^*^	−0.172^**^	−0.076	0.329^**^	−0.269^**^	0.302^**^	1	0.302^**^	0.506^**^	0.440^**^	0.604^**^	0.331^**^	−0.277^**^	0.022	−0.139^*^	0.268^**^	−0.019
Goals	0.040	−0.028	0.049	−0.156^**^	0.041	0.339^**^	−0.157^**^	0.028	0.302^**^	1	0.800^**^	0.445^**^	0.773^**^	0.356^**^	−0.101	0.140^*^	−0.038	0.147^**^	0.158^**^
Impulse	−0.006	0.007	0.042	−0.107	0.005	0.345^**^	−0.213^**^	0.033	0.506^**^	0.800^**^	1	0.477^**^	0.790^**^	0.410^**^	−0.115^*^	0.108	−0.060	0.162^**^	0.156^**^
Non-acceptance	−0.008	−0.011	−0.026	−0.053	−0.040	0.217^**^	−0.134^*^	0.144^*^	0.440^**^	0.445^**^	0.477^**^	1	0.725^**^	0.370^**^	−0.177^**^	0.030	−0.145^**^	0.230^**^	0.075
Strategies	−0.042	−0.019	−0.009	−0.150^**^	−0.024	0.373^**^	−0.236^**^	0.039	0.604^**^	0.773^**^	0.790^**^	0.725^**^	1	0.491^**^	−0.192^**^	0.061	−0.118^*^	0.240^**^	0.138^*^
Anxiety	0.003	0.012	0.031	0.070	0.012	0.116^*^	−0.046	0.070	0.331^**^	0.356^**^	0.410^**^	0.370^**^	0.491^**^	1	−0.074	0.028	−0.027	0.073	0.070
Personal interest	0.355^**^	0.246^**^	0.271^**^	0.351^**^	0.043	−0.214^**^	0.356^**^	−0.116^*^	−0.277^**^	−0.101	−0.115^*^	−0.177^**^	−0.192^**^	−0.074	1	0.190^**^	0.586^**^	−0.712^**^	0.091
Test oriented	−0.021	0.203^**^	−0.052	−0.035	0.158^**^	0.118^*^	0.054	0.032	0.022	0.140^*^	0.108	0.030	0.061	0.028	0.190^**^	1	0.232^**^	0.017	0.212^**^
Vocation oriented	0.112^*^	0.298^**^	0.049	0.050	0.108	−0.188^**^	0.190^**^	−0.036	−0.139^*^	−0.038	−0.060	−0.145^**^	−0.118^*^	−0.027	0.586^**^	0.232^**^	1	−0.533^**^	0.120^*^
Ambivalent	−0.216^**^	−0.183^**^	−0.164^**^	−0.287^**^	−0.040	0.316^**^	−0.339^**^	0.153^**^	0.268^**^	0.147^**^	0.162^**^	0.230^**^	0.240^**^	0.073	−0.712^**^	0.017	−0.533^**^	1	0.035
Procrastination	−0.069	0.088	−0.042	−0.053	0.147^**^	0.157^**^	−0.146^**^	−0.021	−0.019	0.158^**^	0.156^**^	0.075	0.138^*^	0.070	0.091	0.212^**^	0.120^*^	0.035	1
Resilience	0.287^**^	0.165^**^	0.237^**^	0.320^**^	0.064	−0.160^**^	0.452^**^	−0.243^**^	−0.387^**^	−0.373^**^	−0.360^**^	−0.351^**^	−0.490^**^	−0.227^**^	0.306^**^	0.026	0.157^**^	−0.329^**^	−0.049

* *Significant at 0.05 level*.

** *Significant at 0.01 level*.

The procedure for classifying the participants into homogeneous groups, according to their responses to the 20 subscales, has already been described (in section Data Analysis); according to this method, the best solution consists of four clusters, provided by “manhattan” distance and “ward.D” clustering method. Hence, Table 4 provides us with the means of subscales for each cluster and the results of the multiple pair-wise comparisons among clusters (using the Kruskal-Wallis test and function “kruskalmc,” at 0.05 significance level). Note that all the mean values of the 20 subscales used in cluster formulation, are statistically different through the four clusters; furthermore, in eight out of twenty subscales (Deep, Personal Interest, Vocation Oriented, Ambivalent, Clarity, Non-acceptance, Resilience, and Anxiety) the differentiation is quite strong, since five out of six pair-wise comparisons were statistically significant, whereas in two subscales (External and Test Oriented) only one out of six pairs had significant difference. The last two lines of Table 4 refer to the effect of the cluster solution on GPA and success rate; clusters 1 and 3 have the statistically significant highest GPA. Cluster 1 also seems to have the highest success rate whereas the success rate of cluster 3, is not statistically different than this of cluster 4.

**Table 4:** Cluster solution and multiple pair-wise comparisons.

	**Means**				**Pair-wise Comparisons (1: difference is statistically significant at 0.05 level, 0: otherwise)**					
	**1**	**2**	**3**	**4**	**1–2**	**1–3**	**1–4**	**2–3**	**2–4**	**3–4**
	**(*n* = 131)**	**(*n* = 39)**	**(*n* = 63)**	**(*n* = 83)**						
Deep	0.570	−0.505	0.166	−0.788	1	1	1	1	0	1
Stepwise	0.069	−0.568	0.553	−0.261	1	1	0	1	0	1
Concrete	0.492	−0.384	0.190	−0.740	1	0	1	0	0	1
Self	0.538	−0.461	−0.113	−0.547	1	1	1	0	0	1
External	−0.087	−0.315	0.002	0.284	0	0	0	0	1	0
Lack	−0.320	0.805	0.317	−0.113	1	1	0	0	1	0
Personal interest	0.319	−1.119	0.362	−0.253	1	0	1	1	1	1
Test oriented	−0.169	−0.084	0.338	0.051	0	1	0	0	0	0
Vocation oriented	0.054	−0.868	0.575	−0.114	1	1	0	1	1	1
Ambivalent	−0.380	1.078	−0.303	0.323	1	0	1	1	1	1
Self efficacy	0.404	−0.776	−0.048	−0.236	1	1	1	1	0	0
Awareness	−0.234	0.507	−0.191	0.276	1	0	1	1	0	1
Clarity	−0.368	1.251	0.343	−0.267	1	1	0	1	1	1
Goals	−0.476	0.879	0.991	−0.414	1	1	0	0	1	1
Impulse	−0.462	1.113	0.958	−0.521	1	1	0	0	1	1
Non-acceptance	−0.317	1.478	0.265	−0.395	1	1	0	1	1	1
Strategies	−0.504	1.488	0.822	−0.527	1	1	0	0	1	1
Resilience	0.503	−1.084	−0.263	−0.084	1	1	1	1	1	0
Anxiety	−0.348	1.085	0.406	−0.269	1	1	0	1	1	1
Procrastination	−0.321	−0.025	0.563	0.092	0	1	1	1	0	1
GPA	7.248	6.071	7.170	6.680	1	0	1	1	0	1
Success Rate	0.756	0.579	0.663	0.660	1	0	1	0	0	0

Following a non-parametric bootstrap approach through MANOVA, we could further confirm the mean difference for the 20 subscales, among clusters; specifically, the function “MANOVA.wide,” provided by package “MANOVA.RM” in R (Friedrich et al., 2019), return a Wald-Type Statistic equal to 1566.031 (*p* < 0.001) and a modified ANOVA-Type Statistic equal to 2075.464 (*p* < 0.001), and therefore the assumption that the mean vector remains the same through clusters is rejected.

In order to assess the accuracy of the classification into the derived four clusters based on the 20 subscales, we used different methods, as linear or quadratic discriminant analysis and random forest algorithm; it is known that the quadratic discrimination is more robust to departures from multivariate normality (the hypothesis that our data comes from multivariate normal distribution is rejected) and it can also deal with the case of different covariances matrices among clusters (the homogeneity of variances was also rejected; see e.g., Michie et al., 1995). However, for our data set, the numerical experimentation we carried out showed that random forest (see e.g., Breiman, 2001; Liaw and Wiener, 2002) algorithm performs better than the previous two discrimination approaches. Therefore, using a training sample of size 246 cases (then, the remaining 70 cases were used as validation sample) and function “randomForest” in R (Liaw and Wiener, 2002; the number of trees to grow was set to 5,000 while the number of variables randomly chosen was set to 2). Table 5 shows that the overall prediction was accurate in almost 85% of the cases in the training sample, and a similar (~86%) proportion was noted in the validation set; the lowest accuracy found at the members of cluster 2.

**Table 5:** The random forest (classification) algorithm.

		**Predicted cluster**				
		**1**	**2**	**3**	**4**	**Accuracy**
**Training set**						
Actual cluster	1	83	0	7	5	87.37%
	2	2	22	3	4	70.97%
	3	4	3	46	2	83.64%
	4	6	0	2	57	87.69%
**Validation set**						
Actual cluster	1	33	0	1	2	91.67%
	2	0	6	2	0	75.00%
	3	1	0	7	0	87.50%
	4	1	1	2	14	77.78%

It remains of high importance to understand that the adoption of particular learning patterns, students' motivation and non-cognitive characteristics are based on a dynamic interaction between different factors and variables, focusing on the student him/herself. Moreover, the present study takes into account variables from fields that have not been studied in conjunction: resilience, emotion dysregulation and anxiety (mental health field).

The current study answers the question regarding the way in which these specific factors could possibly interact cumulatively, influencing students' pace of study, procrastination and GPA.

Previous studies exploring first-year students' academic success have come up with two to four profiles (Haarala-Muhonen et al., 2017; Lindblom-Ylänne et al., 2017). However, as these studies do not include variables from different theoretical traditions, it would be interesting to explore whether mental health variables could also contribute to the formulation of clusters (Karagiannopoulou et al., 2019). Based on the above we have postulated the following research questions:

RQ1: Which different (meta) cognitive-emotional learner profiles can be identified for first-year students in higher education?

RQ2: How do these different (meta) cognitive-emotional learner profiles differ regarding GPA and success rate?

## Discussion

The aim of the current study was 2-fold: (1) to identify student profiles that include cognitive, metacognitive and motivational aspects of learning, as well as aspects of mental health-resilience, emotion dysregulation anxiety, and procrastination, and (2) to investigate whether or not students with different profiles also differ regarding GPA and success rate in their first year of study. Although current research on students' profiles has identified profiles based on cognitive, metacognitive and motivational aspects of learning, aspects of mental health and well-being have been largely neglected so far (Willems et al., 2018) despite playing a crucial role in students' transition from secondary to higher education and having an impact on students' achievement (Schaeper, 2019). Therefore, the current study focuses on determining (meta)cognitive-emotional learner profiles in first-year students in higher education and how these different profiles differ with regard to academic achievement.

This study distinguished between four different (meta)cognitive-emotional learner profiles (“emotionally stable and highly adaptive learner”; “emotionally dysregulated and at risk learner”; “emotionally dysregulated and highly adaptive learner”; “emotionally stable but at risk learner”), which offers an added value to the already known (meta)cognitive learner profiles presented in previous works. The added value in terms of high GPA scores concerns the role of emotional stability and procrastination, as self-protection factors; concerning the low GPA scores the study raises the issue of emotional instability, whilst raising the flag that some consideration should be given to developmental identity and motivation, implying the need for consultation and mentoring.

In particular, the first profile could be labeled the “emotionally stable and highly adaptive learner” (Cluster 1 in the result section). These students are characterized by applying different cognitive processing strategies, self-regulating their learning process, being interested in learning and highly self-efficacious, being emotionally regulated, resilient, not anxious and not having a tendency to procrastinate their academic work. As described in the literature, this profile demonstrates the positive interaction between self-regulation, self-efficacy, a positive motivation and absence of a tendency to procrastinate (Steel, 2007; Burnam et al., 2014; Katz et al., 2014). This learning profile is considered to be the theoretically preferred profile for students in the first-year of higher education.

The second profile can be labeled as the “emotionally dysregulated and at risk learner” (Cluster 2). The results pointed out that these learners have a low use of cognitive processing strategies, experience lack of regulation, are highly ambivalent motivated, not self-efficacious, emotionally dysregulated, not resilient, anxious and have a tendency toward procrastination. Not surprisingly, students who belong to this learning profile are at risk, both on the emotional and (meta)cognitive aspects of learning. This profile shows that the absence of self-regulated learning goes hand in hand with anxiety (Pintrich, 2004) and emotional dysregulation, but does not come along with procrastination (Chang, 2014).

The third profile can be labeled as the “emotionally dysregulated and highly adaptive learner” (Cluster 3). These students apply different cognitive processing strategies, are mainly externally regulated or lack regulation, are motivated, self-efficacious, emotionally dysregulated, not very resilient, anxious, and have a tendency toward procrastination. It seems that these students are quite good in academic adjustment, meaning that they are adapting their (meta)cognitive learning strategies to the new learning environment (Vermunt, 2005), but are less emotionally adjusted. This highlights the importance of looking at both (meta)cognitive and emotional aspects of students' learning when entering higher education (Schaeper, 2019).

The fourth profile can be labeled as the “emotionally stable but at risk learner” (Cluster 4). These students show extremely low use of cognitive processing strategies, external regulation, ambivalent motivation, and a small degree of resilience. They do to not appear to be anxious and emotionally dysregulated and report only a slight tendency toward procrastination. These learners appear emotionally adjusted but are not academically adjusted to the new learning environment. They lack self-regulation, motivation, and self-efficacy (Katz et al., 2014). Ambivalence and poor self-efficacy that come along with external regulation and lack of regulation may lead to restricted cognitive processing strategies. Possibly, it is not emotional stability that comes along with poor cognition and motivation but the restricted psychological strengths like self-efficacy and resilience that may lead to some level of procrastination.

The study also examined whether the different (meta)cognitive-emotional learner profiles differ regarding success rate and GPA. The 1st and 2nd profile support the relevant literature concerning the links between adaptive and maladaptive profile with achievement (Prosser et al., 2003). Not surprisingly, the students in the “emotionally stable/highly adaptive learner” profile have the highest GPA-score. Adaptive, cognitive, motivational, and emotional factors lead to academic success. Suggest self-regulation and self-efficacy as characteristics of the highly adaptive learner.

Moreover, students in the “emotionally dysregulated and at risk learner” profile have the lowest GPA compared to the “emotional stable and highly adaptive learner,” the “emotionally dysregulated and highly adaptive learner” and the “emotionally stable and at risk learner.” As already described above, the 2nd learner profile is theoretically the least desirable profile and it is also clear that students in this profile also score the lowest GPA. This demonstrates that low self-regulation and low self-efficacy are related to a lower academic achievement (Vermunt, 2005; Komarraju and Nadler, 2013; Burnam et al., 2014) in combination with high levels of emotional dysregulation and anxiety.

Interestingly, students in the 3rd profile report a GPA similar to the adaptive profile. However, these students are procrastinators. Recent literature supports this finding discussing the role of productive procrastination on university students (Westgate et al., 2017). Possibly, procrastination has an adaptive effect on learning for this group of students. It prevents the self from anxiety and emotion dysregulation and enables students to adopt a strong stepwise cognitive strategy to meet test orientation and vocation orientation demands. Surviving anxiety through procrastination enables them to cope effectively with academic demands and to overcome difficulties raised by emotion dysregulation, thus making academic success possible.

Although, the emotionally dysregulated at risk students (2nd profile) report the lowest GPA, similar low achievement scores were reported by the students in the 4th profile that includes emotionally stable students. An interpretation could be that students in the 4th profile may be undecided students without psychological backsets. Poor engagement into any of the cognitive strategies and poor motivation are not followed by emotion dysregulation and anxiety. Some degree of external regulation possibly supports the slightly higher GPA compared to the 2nd profile. Although they do not report emotional deficits that could have a detrimental effect on learning, ambivalence, and poor self-efficacy may prevent them from high achievement. Such a profile may depict identity and developmental issues rather than other problems. Future research should shed light on the psychological and cognitive characteristics of this group of students.

In summary, the two groups of students at risk, one comprising emotionally unstable students (2nd profile) and the other students with poor self-efficacy and ambivalence without any emotional setbacks (4th profile), shed light on previous findings reporting a defensive profile (Karagiannopoulou et al., 2019) and a relaxed profile (Karagiannopoulou et al., 2020), respectively. In the latter, identity issues may illuminate the rather vague picture of the emotionally stable/at risk students. It is suggested that students who score equal on the (meta)cognitive aspects of the learning profile will have comparable GPA-scores regardless of how they score on emotional aspects- mental health variables. However, ambivalence and low self-efficacy seem to draw them back. This is supported by high scores in external-regulation. Setbacks on self-efficacy and self-regulation, main characteristics of highly adaptive learners, may depict developmental issues that could be overcome in the next years (Lindblom-Ylänne et al., 2019). The two low GPA profiles raise the need for different interventions and support to first year students involving counseling and consultation.

Overall, the study (a) supports previous findings about adaptive and maladaptive profiles revealing the contribution of mental health variables in students' learning, (b) shows the role of procrastination as protective factor, (c) raises the need for further exploration of the characteristics of poor achievers who appear emotionally adjusted (4th profile) although with poor characteristics related to learning: self-efficacy, motivation, and self-regulation.

Notwithstanding the positive results above, there are limitations to this study that should be noted. The fundamental limitation associated with this study is that the sample was based solely on undergraduate students at a Greek university. Hence, generalizations to universities in other countries should be made with caution.

Additionally, the issues of culture and diversity are not addressed in the current framework of student engagement. Further research should also look at student engagement predictors and consequences, paying special attention to students' academic performance, health and well-being.

Moreover, some of the limitations are related to the person-oriented perspective adopted in this study. A learner profile combines students with a comparable score on the different scales, but this does not mean that students within a certain profile have the same scores on the different scales included in the profile analysis. It is possible that students find themselves at the “border” of a learner profile and sometimes are more closely related to another learner profile. Not only would it be informative to look at profile membership for diagnostic reasons, but it could also be interesting to look at the differences between the individual scores of a student compared to the mean of the learner profile when diagnosing students.

For future research, we suggest replicating the findings of this study with other datasets in order to control whether the same four profiles can be detected. In addition, it would be interesting to explore outcome variables other than GPA. More specifically, future research could investigate well-being (Trautwein and Bosse, 2017) and university dropout rate using a longitudinal research design.

## Data Availability Statement

The raw data supporting the conclusions of this article will be made available by the authors, without undue reservation.

## Ethics Statement

The studies involving human participants were reviewed and approved by University of Ioannina. The patients/participants provided their written informed consent to participate in this study.

## Author Contributions

EK developed the research proposal, suggested the literature review and the methodology and contributed significantly to the final version of the discussion. CR carried out the literature review and wrote the Introduction and the Methodology sections. The Data Analysis and the Results section were conducted by FM. The first draft of the Discussion was written by LC and DG who significantly contributed to the revised version. The editing and preparation of the manuscript for publication were conducted by SM and CL. All authors supervised the manuscript.

## Conflict of Interest

The authors declare that the research was conducted in the absence of any commercial or financial relationships that could be construed as a potential conflict of interest.
